# Resistance to treatment in eating disorders: a critical challenge

**DOI:** 10.1186/1471-244X-13-282

**Published:** 2013-11-07

**Authors:** Secondo Fassino, Giovanni Abbate-Daga

**Affiliations:** 1 Eating Disorders Center for Treatment and Research, Department of Neuroscience, University of Turin, Via Cherasco 15, Turin 10126, Italy

## Abstract

The Special Issue “Treatment resistance in Eating Disorders” gathers together the contributions provided by several experienced groups of researchers in the field of Eating Disorders (EDs). The main topic is addressed from multiple perspectives ranging from pathogenesis (including developmental and maintaining factors) to treatment. An explicative model of resistance in EDs is also proposed.

## 

The mind commands the body, and it obeys instantly; the mind commands itself, and is resisted [...]. And therefore are there two wills, for that one of them is not entire: and what the one lacketh, the other hath. (Augustine of Hippo AD 398; Confessions)

Challenging resistance to treatment has now become crucial in psychiatric clinical practice also because of the relevance of this topic from a public health standpoint. In fact, research has recently highlighted that other psychiatric illnesses like schizophrenia, depression and bipolar disorders, obsessive-compulsive disorders, and personality disorders are often characterized by either a lack of effect or a moderate response to treatments [1].

Eating Disorders (EDs) are serious and difficult to treat mental illnesses, often showing ego-syntonic features and resistance to treatments; as a result, a chronic course of illness occurs in a considerable number of affected individuals [2]. To date there is a lack of evidence-based treatments effectively impacting the ED outcome [3,4] and therapy is often hampered by the well-known phenomenon of dropout [5].

At the onset of the ED, affected individuals are only rarely aware of their illness and such an attitude can be maintained until death [6]. Some of the aspects contributing to denial and resistance to treatments can be automatic and unmotivated whilst others are consciously generated by patients who actively oppose to treatment, also on the basis of the biological vulnerability of altered reward and inhibition underlying these disorders [7,8].

The ED field could represent an example of both crisis of psychiatry and its moderate effectiveness [9,10] with reductionist approaches also playing a role in this regard [11]. Hence new complex models that take into account the intertwined link between psychology and its biological underpinnings are necessary to better understand these disorders. For example, more emphasis should be placed on the neurobiology of interpersonal relationships and on how treatments can modify the brain from both individual and evolutionary perspective [12].

In the framework of resistance to treatments in EDs, the psychodynamic model suggested in the past by A. Adler, H. Kohut, and D. W. Winnicott seems to be reconceptualized by recent findings, with the EDs emerging in the present articles as disorders of the development of the Self, as already theorized [13,14]. Prompted by earlier lines of research, studies have recently focused on several aspects including: neurobiological effects of attachment and primary relationships, complex gene-environment interaction, temperament and deficits in the development of the character, cognitive inflexibility, and those pseudo-positive meaning that patients attribute to their behaviors and symptoms.

Figure 1 illustrates the EDs as developmental disorders and the vicious cycle that is elicited and maintained by the “ED identity”, potentially a crucial issue in generating resistance. Disorientation and distress that characterize patients since childhood and that are then triggered during adolescence have been graphically emphasized. The biopsychosocial factors that interfere with a mature development of the Self and those aspects that hamper an adaptive personality development are also highlighted. This approach suggests a distinction between mere eating symptomatology (as conceptualized by the Diagnostic and Statistical Manual criteria) and the profound nature and meaning of these disorders. In line with this approach, the ED symptomatology can be severe and overwhelming but the triggering core of the disorder could be represented instead by a primary and multifaceted deficit of personality development. During adolescence, such a deficit could be involved in generating eating symptomatology as both signal of suffering and harmful attempt to self-cure. The process/vicious cycle is graphically represented as reinforced by contaminant emotions [15]: conscious and unconscious meanings, family dynamics, starvation, countertransference and therapist’s reactions, anger, and aggressiveness; all these factors can lead to a chronic course of the disorder.

**Figure 1:**
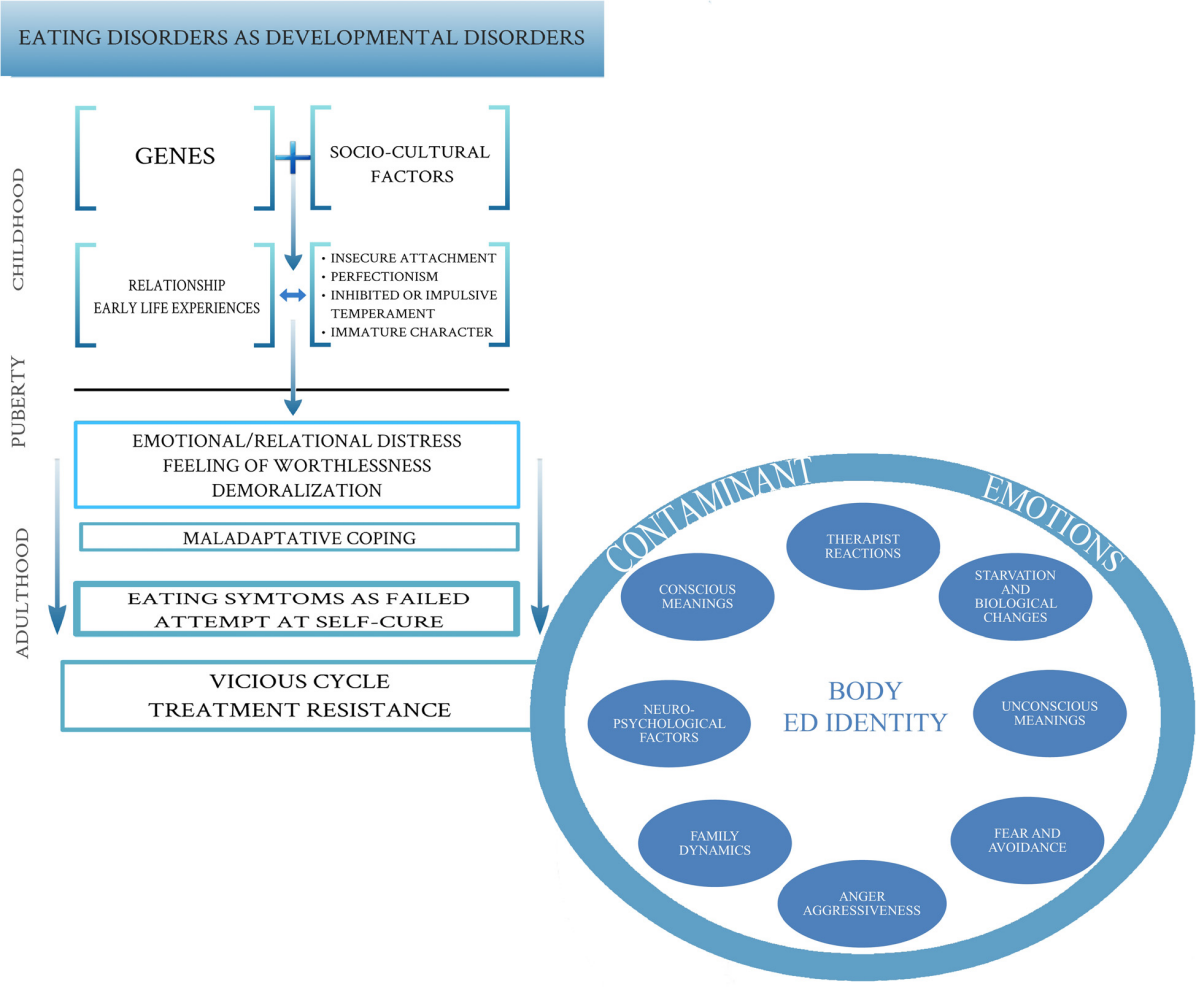
The vicious cycle of treatment resistance in eating disorders.

In other words, such an impaired development could lead to eating symptomatology as a pseudo-adaptive response to profound worthlessness [13,16,17] and to the inability to integrate inner states and external stimuli. This feeling of inferiority is then exaggerated by the illness, with starvation and splitting and obsessive thoughts reinforcing patients’ perfectionistic premorbid traits. Therefore, relational and decision-making abilities are further impaired along with feelings of anger and loneliness.

Within this framework, eating symptomatology mirrors inner ambivalence: on one hand eating behaviors indicate severe psychological discomfort and on the other hand dangerously mask the conflicts that typically occur during adolescence (dependence-independence, gender identification, creativity-shame, etc.). A vicious cycle leading to avoidance can be generated by two aspects: the first is the need to control hunger and weight as a means of regaining self-esteem, and the second is represented by the fear of losing control on eating, life, and emotions [18].

Therefore, from a clinical standpoint, the understanding of the adaptive value of eating symptomatology is crucial to help clinicians considering those needs and conflicts underlying the disorder and to plan shared treatment interventions and goals. An innovative approach to psychiatry should encourage patients to use their own resources to change, hence entailing lower distress in therapy [9]. Moreover, treatment should help patients understand what underlies their illness and also provide alternative motivations and strategies to give up the disorder. In line with treatments for other mental disorders, also in the ED field psychotherapists should encourage patients to use understanding and kindness toward themselves and others as a way to overcome the burden of emaciation, negative emotionality, and avoidance [12].

Since resistance can be also considered as a regulator of the therapeutic relationship, therapists should address this issue being aware of transference and countertransference, and carefully managing their own emotions in addition to patients’ ones [9]: in order to do this, a constant revision of treatment plan and strategies is needed. As noted above, the path to recovery is difficult for those affected by EDs and it should be considered in the wide context of development. Only within a solid therapeutic relationship (i.e. embodied simulation and intentional attunement [19]), a new and mature identity can be acquired, progressively replacing the “ED identity”.

All articles included in this Special Issue provide a useful contribution to shed light on resistance to treatment in EDs. Halmi [20] acknowledged the importance of severity of the ED psychopathology in resistance and Segura-Garcia and Coworkers [21] investigated the role of personality on long-term prognosis. The relevance of Axis I [22] and substance use [23] comorbidities in predicting resistance has been also addressed. It has been then emphasized the usefulness of a comprehensive multidimensional evaluation (i.e. including the assessment of quality of life) for those patients who are resistant to treatment [24] also considering their stages of change to predict their work alliance abilities [25]. Moreover, emotive and perceptual components have been also investigated: individuals recovered from Anorexia nervosa partially maintain altered emotional facial expressions, mainly as regards positive emotions [26]. Data on how to manage resistance to treatments have also been reported by a clinical overview of the available literature on this topic highlighting four main thematic areas: denial, motivation to change, maintaining factors and treatment outcome, and therapeutic relationship [27]. A multicenter study conducted in UK showed that confidence to change, social functioning, and carers’ both expressed emotion and control can predict less resistance, emphasizing the role of family [28]. One contribution advocated the importance of reducing dropouts showing that completion of treatment can predict improved response in Binge Eating Disorder [29] and the role of attention deficit hyperactivity disorder in obese individuals has been debated [30]. Some preliminary results on a modified version of Dialectical Behavioral Therapy focusing on EDs have been also presented [31]. Finally, Marzola et al. [32] underlined the need for evidence-based data on nutritional restoration in Anorexia nervosa in order to further the understanding of the pathophysiology of this disorder and provide clinicians with clear guidelines to minimize resistance.

We believe that BMC Psychiatry can successfully accomplish a wide diffusion of these data and also improve the debate on the topic of resistance to treatment in EDs. The latter is indeed relevant in clinical practice and controversial in research. BMC Psychiatry with its comprehensive framework can strongly help us in this challenge since it is an open access, popular, and peer-reviewed journal. BMC Psychiatry values the psychosomatic approach since it considers articles on all aspects of psychiatric disorders: prevention, diagnosis and clinical management.

So what are the conclusions?

In the ongoing debate on treatment resistance in EDs several aspects remain controversial. If the interplay between genes and environment remains highly unclear, from a therapeutic point of view the main issue then becomes the need of an authentic and solid alliance, particularly with those patients who are resistant to treatment. A major caveat is represented by the need of avoiding collusion or sharp disagreements with patients keeping in mind that the overarching goal is to minimize the risk of therapy failure. The primary aim is to reassure patients that the vital and compensatory aspects of their psychopathology have been deeply understood and their suffering will be carefully considered while planning treatment interventions. Moreover, it is important to measure and compare the expected changes with patients’ ability to tolerate distress, minimizing in this way the risk of dropout. As Augustine of Hippo stated many centuries ago, the human mind is conflicting itself, and those affected by an ED represent a paradigm of the conflict between mind and body and life and death, causing often anxiety, helplessness, and compassion in therapists. It is noteworthy that Anorexia nervosa remains an enigma: those symptoms that impair life and body to the point of death can also alleviate inner discomfort. Only a strong knowledge and a firm empathic approach – enhancing a secure attachment – can successfully help therapists to be effective and limit patients’ omnipotent and destructive desperation.

What’s next? We hope that this special issue can encourage future work on three major lines of research on resistance to treatment in EDs: a) biological factors, b) emotions in affected individuals and their families, and c) therapeutic relationship.

Concluding with particular reference to therapeutic alliance, we would like to report here what A. Adler wrote years ago: “I cannot forget what one of my cured patients once answered when I asked him: “What do you believe was the reason that I could succeed to cure you after all these years of misery?”. He answered: “I became sick because I had lost all hope. And you gave me hope” [33].

## Competing interests

The authors declare that they have no competing interests.

## Pre-publication history

The pre-publication history for this paper can be accessed here:

http://www.biomedcentral.com/1471-244X/13/282/prepub
